# Risk-based contracting for high-need Medicaid beneficiaries: The Arkansas PASSE program

**DOI:** 10.1016/j.hpopen.2020.100023

**Published:** 2020-12-03

**Authors:** Adrienne Nevola, Michael E. Morris, Carrie Colla, J.Mick Tilford

**Affiliations:** a Department of Health Policy and Management, University of Arkansas for Medical Sciences, 4301 West Markham, #820, Little Rock, AR 72205, United States; b Department of Health Policy, Economics, and Management, University of Texas Health Science Center, 11937 U.S. Highway 271, Tyler, TX 75708, United States; c The Dartmouth Institute for Health Policy and Clinical Practice, Geisel School of Medicine, 1 Medical Center Drive, Lebanon, NH 03756, United States

**Keywords:** Mental disorders, Developmental disabilities, Payment reform, Provider-sponsored organizations

## Abstract

The high cost of health care for people with behavioral health (BH) conditions or intellectual and developmental disabilities (IDD) in the United States led one state to implement the Provider-led Arkansas Shared Savings Entity (PASSE) program. PASSE is a managed care model that puts provider-led organizations at risk for the highest need people with BH conditions or IDD in Medicaid, a public health insurance program for low-income residents. Drawing on key informant interviews and payment models across the United States, we describe the PASSE program, how it compares with state Medicaid programs for similar populations, and prospects for the program. Key informants cited several PASSE features as promising mechanisms to improve beneficiary outcomes: expanded care coordination, service flexibility, incentives for community investments, accountability for cost and quality across physical health, behavioral health, and long-term care, quality target accountability, fostered competition, and provider ownership. Informants worry that PASSE features will be insufficient to catalyze changes in provider behavior. Efforts may be targeted to control costs primarily through service reductions with uncertain effects on quality of care. PASSE hinges on improved care coordination and increased efforts to address social determinants of health. Success or failure in these areas will likely determine whether PASSE leads to improved outcomes for two of the most costly and vulnerable populations.

## Highlights

• Healthcare for people with behavioral health conditions or developmental disabilities is costly.
• One state in the U.S. is testing a provider-led, capitated payment model.
• The model serves low-income, high-need people with BH conditions or DD.
• The model may improve care coordination, lower costs, or limit services.
• The narrow population focus may hinder changes in provider behavior.

## 1 Introduction

Medicaid is a health insurance program in the United States for people with low income and for those with disabilities. Each state operates a Medicaid program, which is jointly funded by the state and the federal government. Medicaid consumes an ever-growing share of state budgets, crowding out other state spending obligations like education and infrastructure. From 1988 to 2014, the percent of state funds devoted to Medicaid rose from 8.7 percent to 19.3 percent [1]. Congress and state legislatures are tackling the growth in Medicaid spending with policies that reduce enrollment, reimbursement rates, or utilization [2], and often target high cost services or populations. Beneficiaries with behavioral health (BH) conditions or intellectual and developmental disabilities (IDD) are two such populations. Almost half of Medicaid spending goes toward the care of the 20 percent of Medicaid beneficiaries with BH conditions [3]. Individuals with IDD are a similarly small but costly set of Medicaid beneficiaries [4]. For home and community-based services, a growing Medicaid service alternative to institutionalization, beneficiaries with IDD account for 40 percent of enrollees but 70 percent of spending [5]. Policies that aim to reduce spending are increasingly directed at these two high-cost populations.

By design, Medicaid covers people with disabilities and those with low income and resources. The poverty rate of people with disabilities is more than twice that of people without disabilities [6]. The high rate of poverty and disability status of people with BH conditions or IDD results in Medicaid playing an outsized role in funding care for these two populations. It covers 26 percent of adults with serious mental illness compared to only 14 percent of the general adult population [3], and funds 78 percent of services and supports for people with IDD [7]. The disproportionate role of Medicaid as the insurer for people with BH conditions or IDD heightens the positive or negative impact it can have on the health and quality of life of these populations. Faced with the escalating cost of care and consistently poor outcomes for people with BH conditions or IDD [8], [9], [10], [11], states are testing new approaches for Medicaid beneficiaries with these conditions to control spending or improve quality.

Arkansas follows the national trend in devoting a disproportionate share of Medicaid spending – 31 percent, or $2 billion in 2017 - to the 14 percent (~150,000) of beneficiaries with BH conditions or IDD [12], [13]. In December 2015, Arkansas Governor Asa Hutchinson called for a reduction in the cost of Medicaid by $835 million over five years [14], [15]. Over the next year the Governor, the Department of Human Services (DHS) and the bipartisan Health Reform Legislative Task Force examined possible reforms that would make the Arkansas Medicaid program sustainable for the future. The task force initially considered Health Homes, a model of care coordination for Medicaid beneficiaries with serious mental illness, but dropped the initiative due at least in part to provider resistance [16]. Two payment models eventually emerged: (1) to contract with a small number of full-risk managed care organizations, or (2) to operate a managed fee-for-service model through an administrative service organization. In the last quarter of 2016, DHS conducted a series of public meetings to assess the potential for developing a hybrid of the two proposals, borrowing advantages from each model. Under the hybrid model, Arkansas would merge provider leadership with the tools and risk-bearing expertise of managed care organizations [13]. During the winter of 2016–2017, interest and support among providers and their potential partners grew, leading to the introduction of H.B. 1706, ‘To Create the Medicaid Provider-Led Organized Care Act’ in the 91st Regular Session. The Senate and House each passed the Act as amended with just one dissenting vote. Governor Hutchinson signed Act 775 into law on March 31, 2017.

Through the provision of care coordination and the alignment of provider incentives, the Provider-led Arkansas Shared Savings Entity (PASSE) program aims to integrate physical, behavioral, and long-term care for high-need Medicaid beneficiaries with BH conditions or IDD. PASSE beneficiaries have high functional needs due to the severity and complexity of their disabilities, and reside in a high poverty, predominately rural state, making PASSE accountable for the care for one of the most vulnerable populations in the country. Our goals are to describe PASSE, detail how its principal features compare to other state Medicaid programs for people with BH conditions or IDD, discuss its development, and provide key stakeholder views of the prospects for the program.

### 1.1 PASSE features

The Medicaid Accountable Care Organization Learning Collaborative suggests state Medicaid agencies address a set of key questions when designing their accountable care programs [17]. Organized by key question, we describe the features of PASSE below.

#### 1.1.1 Whom does PASSE cover?

Arkansas recognized that ~ 28,000 of the ~ 150,000 beneficiaries with IDD or BH conditions accounted for $1.1 billion annually, or half of the spending for this group. To capture the beneficiaries in this ‘tail of the spending distribution’ without using historical spending directly, the state defined the mandatory population as beneficiaries with IDD or BH conditions with the highest levels of functional need. To our knowledge, no other state has designed a risk- or value-based Medicaid model around only the most complex BH or IDD beneficiaries. To identify the subset of beneficiaries with the highest needs, the state contracted with an independent organization to conduct a functional assessment of all BH and IDD beneficiaries. The assessment categorized beneficiaries into one of three tiers (see Table 1). Beneficiaries falling into the two higher needs tiers comprise the mandatory PASSE population. Tier I beneficiaries may have the option to enroll in PASSE in the future.

**Table 1:** PASSE Tier Assignment Criteria.

		**BH population**	**IDD population**
**NoExcluded fromPASSE enrollment**	**Tier I**	Counseling Level Services Time-limited behavioral health services are provided by qualified licensed practitioners in an outpatient-based setting for the purpose of assessing and treating mental health and/or substance abuse conditions. Counseling services settings include behavioral health clinics/offices, healthcare centers, physician offices, and/or schools.	Community Clinic Level of Care At this level of need, the individual receives services in a center-based clinic such as a developmental day treatment clinic services (DDTCS) or child health management services (CHMS).
**Mandatory PASSE enrollment**	**Tier II**	Rehabilitative Level Services Services are provided in a counseling services setting but the level of need based on the independent assessment requires a broader array of services to address functional deficits.	Institutional Level of Care The individual meets the institutional level of care criteria but does not need care 24 h a day and 7 days a week.
	**Tier III**	Intensive Level Services Eligibility will be identified by additional criteria and questions derived through the independent assessment which could lead to placement in residential settings for more intensive delivery of services	Institutional Level of Care 24/7 The individual meets the institutional level of care and requires care 24 h a day and 7 days a week.

#### 1.1.2 What services does PASSE provide?

Each PASSE is accountable for the physical, behavioral, and long-term care of its attributed population; only a few services and settings, such as non-emergency medical transportation and skilled nursing facilities, are excluded. In accordance with the 2016 Medicaid managed care regulations [18], [19], PASSE adopted an expanded definition of care coordination that requires coordination across clinical and nonclinical services. PASSEs may employ or contract with care coordinators, who are responsible for the promotion of activities focused on the health of the beneficiary and their community, including developing and monitoring a person-centered service plan, coordinating and referring to services including medication management, providing health education and coaching, and assisting with social determinants of health. The program also capitalizes on the revised managed care regulations by allowing PASSEs to cover services (e.g., equine therapy) that would be prohibited under fee-for-service reimbursement. The services may take the form of “in-lieu-of” or “value-added” services depending on whether they are included in the capitation rate, but either way, PASSEs are permitted to count the expenditures on these approved services as member costs (as opposed to administrative costs) when calculating their medical loss ratio. PASSEs may also make investments in the community, consistent with 45 §158.150 “Activities That Improve Health Care Quality” and count these investments as member costs up to an amount equal to 5 percent of revenue. The expanded care coordination definition, and the permission to treat approved services not covered under Medicaid fee-for-service and a portion of community investments as member costs create opportunities and incentives to provide appropriate and effective services, including those targeted to the social needs of beneficiaries such as housing and employment [20].

#### 1.1.3 How are beneficiaries attributed?

Each PASSE is required to provide statewide coverage. Initial attribution of Tier II or III beneficiaries to a PASSE was based on the prior relationship between the beneficiary and his or her chief provider, and was designed to favor relationships with BH or IDD providers. Beneficiaries could select a different PASSE ninety days after this initial assignment, and at the beginning of each year thereafter. As of December 2018, approximately 46,000 individuals were assessed as Tier II or III and were enrolled in the PASSE program; most (approximately 38,000) were eligible due to a BH condition. Of the beneficiaries with IDD, about 60 percent (~4,600) were enrolled in the existing DD Medicaid waiver, about 30 percent (~2,400) were on the waiting list for the waiver, and about 10 percent (~750) resided in privately owned institutions [21]. About 70 percent of PASSE beneficiaries are children; approximately 13 percent are dually eligible for Medicare. Moving forward, new beneficiaries will be offered a choice of PASSEs, and in the absence of a choice will be auto-assigned to the active PASSEs (i.e., PASSEs without sanctions) in a round-robin fashion.

#### 1.1.4 How is the PASSE payment model structured?

Each PASSE receives a global, monthly per-beneficiary capitation payment, adjusted for beneficiary age (child or adult), diagnostic eligibility category (BH, IDD, or dually diagnosed), Medicare eligibility (Medicaid-only or Medicare/Medicaid dual eligible), tier level, and region of the state (north or south) [22]. True to the *shared savings* in the program name, the PASSEs will retain any savings generated or absorb any losses sustained within a certain threshold, and will share in the savings or losses beyond that threshold (Table 2) [22].

**Table 2:** PASSE Risk Corridor.

**Medical Loss Ratio Claims Corridor**	**PASSE Share of Gains / Loss in Corridor**	**DHS Share of Gain / Loss in Corridor**
<81.5%	0%	100%
81.5% to 91.5%	50%	50%
91.5% to 93.5%	100%	0%
93.5% to 98.5%	50%	50%
>98.5%	0%	100%

As a managed care entity, each PASSE will pay an annual 2.5 percent premium tax. The state plans to use the premium taxes for two purposes: (1) to increase enrollment by reducing the IDD waiver waiting list, and (2) to incentivize improved beneficiary outcomes through payment of financial rewards to the PASSEs. PASSEs will be monitored on measures of access (e.g., ratio of beneficiaries to care coordinators and to providers), satisfaction (PASSEs are required to administer beneficiary satisfaction surveys and report the results to DHS), process (e.g., HEDIS® measures of timeliness of follow up care after hospitalization and after new a prescription for attention-deficit/hyperactivity disorder), and utilization (e.g., emergency department use, avoidable hospitalizations) commonly used in outsourced Medicaid programs [23]. DHS has indicated an intention to rely heavily on outcome measures in assessing the success of PASSE and in decisions about the distribution of shared savings and quality incentive payments [13]. Some outcome measures, including patient-reported measures such as the National Core Indicators for beneficiaries with IDD and Consumer Assessment of Healthcare Providers and Systems (CAHPS) metrics have been specified, but much work remains to identify or craft quality metrics, to determine which metrics will factor into the distribution of incentive payments, to establish performance targets, to collect the data needed for the agreed-upon measures, and to assign and enforce both DHS and PASSE accountability for meeting the performance targets.

#### 1.1.5 Who leads the PASSEs?

An IDD provider, a BH provider, a hospital, a physician, and a pharmacist together must own at least 51 percent of each PASSE, and partner with an organization experienced in insurance administration. PASSEs are required to recruit statewide networks of diverse provider types, and may negotiate a variety of payment agreements (e.g., value-based incentive payments, shared savings arrangements), which are expected to promote cost reduction and quality improvement. DHS allows and encourages providers who are not equity owners in a PASSE to join the network of each PASSE and to enter into alternative payment arrangements with one or more PASSEs. PASSEs must determine how to assess the quality of network providers as well as which metrics will factor into shared savings or incentive payments to providers with whom PASSEs have entered into alternative payment agreements.

### 1.2 PASSE compared to other state Medicaid programs

Among state Medicaid programs in general, there is considerable variation in eligibility criteria, coverage policy, payment rates, and other structural elements. Except for covering only the highest need beneficiaries, PASSE shares several features in common with other state Medicaid programs for people with BH conditions or IDD. To better understand the array of programs for these two populations, we conducted a review of Medicaid state agency websites, managed care and accountable care contracts, approved waivers, program evaluations, and other publicly available sources. We first identified the programs in which an entity other than the state Medicaid agency is accountable for the physical, behavioral, and long-term quality and cost of care of its attributed population. For each of these programs, we outline the degree of similarity to PASSE, namely (1) beneficiary attribution (2) payment, and (3) leadership.

Other than PASSE, 29 programs in twenty-six states shift accountability, for both the physical and behavioral health of the BH population to an entity other than the state Medicaid agency (see Table 3). Like PASSE, beneficiaries in 20 programs may choose the accountable entity. For one additional program (ACOs in Massachusetts) beneficiary choice depends on the model. Seventeen programs require statewide coverage. Almost all (24 programs) entities receive capitation payments, with payment tied to performance in approximately two-thirds (19) of programs. Only seven programs were required to be provider-led.

**Table 3:** State Medicaid Programs in Which An Entity Other Than The State Medicaid Agency Is Accountable for the Physical and Behavioral Health Quality and Cost of Care of the BH Population.

		**Beneficiary Attribution**		**Payment**		**Leadership**
**St.**	**Program Name**	**Beneficiaries choose accountable entity?**	**Entity responsible for statewide coverage?**	**Payment structure**	**Performance tied to payment?**	**Entity required to be provider-led?**
AZ	*Arizona Health Care Cost Containment System (AHCCCS), Integrated Care*[74], [75]	No	No	Capitation	AZ free to do this per contract	No
AR	*Provider-led Arkansas Shared Savings Entity (PASSE)*[13]	Yes	Yes	Capitation & shared savings/ losses	Yes	Yes
CO	*Accountable Care Collaborative*[51], [76]	No	No	Care coordination fee & P4P (physical health); Capitation (BH)	Yes	Yes
CT	*Person Centered Medical Homes Plus (PCMH + )*[51], [77]	No	Yes	Shared savings	Yes	Yes
FL	*Magellan Complete Care Specialty Plan*[78], [79]	No	No	Capitation	Yes	No
IL	*Integrated Care Program (ICP)*[80], [81]	Yes	No	Capitation	Yes	No
IA	*IA Healthlink*[82]	Yes	Yes	Capitation	Yes	No
KS	*KanCare*[83]	Yes	Yes	Capitation	Yes	No
KY	*Kentucky Medicaid Managed Care*[84]	Yes	Yes	Capitation	No	No
LA	*Healthy Louisiana*[85]	Yes	Yes	Capitation	Yes	No
ME	*Accountable Communities* (ACOs)[51], [86]	No	Yes	Shared savings/ losses	Yes	Yes
MA	*Managed Care Organizations* (MCOs); *Accountable Care Partnership Plans* (ACOs, 3 models)[51], [87], [88], [89]	Yes for MCOs; Depends on model for ACOs	Yes for MCOs; No for ACOs	Capitation or shared savings/ losses	Yes	No for MCOs; Yes for ACOs
MN	*Prepaid Health Plans/Managed Care Organizations (MCOs)*; *Integrated Health Partnerships* (ACOs)[51], [90], [91]	Yes for MCOs; No for ACOs	No for MCOs; Yes for ACOs	Capitation for MCOs; Global fee or shared savings/ losses for ACOs	Yes	No for MCOs; Yes for ACOs
MS	*MississippiCAN*[92]	Yes	Yes	Capitation	MS free to do this per contract	No
NE	*Heritage Health*[93]	Yes	Yes	Capitation	Yes	No
NH	*New Hampshire Medicaid Care Management*[94]	Yes	Yes	Capitation	Yes	No
NM	*Centennial Care*[95], [96]	Yes	Yes	Capitation	Yes	No
NY	*Health and Recovery Plans (HARP)*[97]	Yes	No	Capitation	Yes	No
OH	*Medicaid Managed Care Program*[98], [99]	Yes	No	Capitation	Yes	No
OR	*Coordinated Care Organizations (CCO)*[51], [100]	Yes	No	Capitation	Yes	Yes
RI	*Comprehensive Accountable Entities* (AEs); *RiteCare* (MCOs)[51], [101], [102], [103]	Yes for MCOs; No for AEs	Yes	Capitation for MCOs; Shared savings/ losses for AEs	Yes	No for MCOs; Yes for AEs
SC	*Healthy Connections*[104]	Yes	Yes	Capitation	Yes	No
TN	*TennCare*[105], [106]	Yes	No	Capitation	Yes	No
VT	*Global Commitment to Health*[107], [108]	No	Yes	Capitation	No	No
VA	*Commonwealth Coordinated Care (CCC) Plus*[109], [110]	Yes	Yes	Capitation	*Information not found*	No
WA	*Integrated Managed Care*[111]	Yes	No	Capitation	WA free to do this per contract	No
WV^a^	*Mountain Health Trust*[112]	Yes	Yes	Capitation	No	No

a Opioid use treatment waiver services and children’s residential treatment facilities are excluded.

Only Arizona, Iowa, Kansas, Maine, and Vermont like Arkansas have shifted accountability for the physical health, behavioral health, and long-term services and supports for their IDD populations (see Table 4). Like Arkansas, Iowa and Kansas allow beneficiaries to choose their own accountable entity, but only Arkansas and Maine require the entity to be provider-led. All but one (Maine) of these states are capitated models with four of six tying performance to payment. In all six states, each entity is responsible to provide statewide coverage. No state administers a program identical to PASSE in beneficiary attribution, payment, and leadership for either the BH or IDD population.

**Table 4:** State Medicaid Programs in which an Entity Other Than the State Medicaid Agency Is Accountable for the Physical, Behavioral Health, and Long-Term Services and Supports Quality and Cost of Care of the Intellectual and Developmental Disabilities Population.

		**Beneficiary Attribution**		**Payment**		**Leadership**
**St.**	**Program Name**	**Beneficiaries choose accountable entity?**	**Entity responsible for statewide coverage?**	**Payment structure**	**Performance tied to payment?**	**Entity required to be provider-led?**
AZ	*Arizona Health Care Cost Containment System (AHCCCS), Arizona Long Term Care System*[113], [114]	No	Yes	Capitation	No	No
AR	*Provider-led Arkansas Shared Savings Entity (PASSE)*[13]	Yes	Yes	Capitation & shared savings/ losses	Yes	Yes
IA	*IA Healthlink*[82]	Yes	Yes	Capitation	Yes	No
KS	*KanCare*[83]	Yes	Yes	Capitation	Yes	No
ME	*Accountable Communities* (ACOs)[86]	No	Yes	Shared savings/ losses	Yes	Yes
VT	*Global Commitment to Health*[107], [108]	No	Yes	Capitation	No	No

While not equivalent in design, Arkansas and other states with recently implemented managed care models for people with complex needs[24] s can still learn from the outcomes found in similar state Medicaid programs. The accountable care organization (ACO) model is similar to PASSE in many ways, including accountability for cost and quality of care. While ACOs have struggled to integrate BH into their care coordination models [25], early evidence indicates ACOs that implement integrated care models are more likely to achieve both cost savings and quality improvement [26], suggesting positive potential for PASSE. An evaluation of the first three years of Oregon’s Medicaid ACOs (coordinated care organizations, or CCOs) found decreased per-person spending, and quality improvements in increased preventive care for children and adolescents, reduced emergency department use, avoidance of low-value care, and improved self-reported health [27]. Some quality measures, though, such as care for people with chronic conditions, did not show improvements, and access to care worsened, perhaps due in part to the large uptick in enrollment from Medicaid expansion in 2014 [27]. CCOs still struggle in some areas, such as integrating physical and behavioral health care, incorporating alternative payment models into provider agreements, and addressing social determinants of health [27]. Barriers to integrating physical and behavioral health care include difficulty restructuring contracts in place prior to CCO implementation, disparities in reimbursement levels for licensed and unlicensed BH providers, and contradictory federal and state guidance on the requirements to pay for non-billable services [28]. Minkoff suggests five areas of focus to integrate physical and BH services: alignment of financial incentives with desired outcomes, enhanced data collection and sharing between physical and BH care delivery systems, enacting legislative and policy changes to remove regulatory barriers, better use of BH quality measures, and alignment with existing initiatives such as patient-centered medical homes [29].

Three programs for beneficiaries dually eligible for Medicare and Medicaid also aim to integrate acute and long-term care for a vulnerable and costly population. The Program of All-Inclusive Care for the Elderly (PACE) began in the 1980 s as a demonstration, and was permanently authorized in 1997. Through a single three-way contract between the Centers for Medicare and Medicaid Services (CMS), the state Medicaid agency, and the operating organization, PACE uses capitation payments to serve individuals age fifty-five and over, primarily dually eligible beneficiaries, who are eligible for nursing facility care. A *meta*-analysis of PACE evaluations suggested the program was associated with fewer inpatient hospitalizations and a lower mortality rate, but at the cost of increased Medicaid spending, primarily due to higher rates of institutionalization [30]. The remaining two programs are not limited to beneficiaries eligible for nursing facility care. Dual-eligible special needs plans (D-SNPs) are Medicare Advantage (MA) plans limited to beneficiaries dually eligible for Medicaid. First offered in 2006 and permanently authorized in 2018, D-SNPs use separate contracts with CMS and with the state Medicaid agency to integrate care for dually eligible beneficiaries under capitated payment arrangements. The most recent CMS demonstration to integrate care for dually eligible beneficiaries, the Financial Alignment Demonstration, uses a three-way contract between CMS, the state Medicaid agency, and the Medicaid-Medicaid Plan (MMP) to integrate care. Importantly, the financial alignment demonstration allows states to share in any Medicare savings achieved by MMPs, while D-SNPs do not. MMPs may receive capitation payments or operate under a managed fee-for service model. The 13 participating states tested 3 to 5-year demonstrations, which ended as early as 2017 or are scheduled to end in 2020. Preliminary findings outlined in a 2018 report from the Medicare Payment Advisory Commission showed MMPs struggled with some of the same implementations issues as the PASSEs, such as locating beneficiaries to conduct need assessments [31]. Interviews with key stakeholders and early evaluations of administrative data find a decrease in emergency department, inpatient, and nursing facility care. Plans recognize the need to address social determinants of health, and are working to develop relationships with local social service resources. CAHPS patient experience for the first three years of the demonstration indicate MMPs perform on par with other MA plans and fee-for-service Medicare. HEDIS® clinical quality measures for the first two years show mixed results, with MMPs tending to perform better than other MA plans on measures included in their quality withhold [31]. Enrollment in MMPs has been lower than anticipated. While the use of passive enrollment (where the beneficiary is automatically enrolled but retains the right to opt out) has been associated with higher participation rates, how passive enrollment is implemented and administrative issues such as inaccurate contact information for beneficiaries and poor outreach materials may limit the usefulness of this enrollment method [32], [33]. DHS may look to lessons learned from MMP enrollment when PASSE is opened to tier I beneficiaries.

## 2 Methods

The success of the PASSE program rests largely with the provider-led organizations, and beneficiaries and other providers of the program. Their experience as PASSE is implemented, and their perspective on the potential benefits and concerns with PASSE may influence their behavior and thus the outcomes of this innovative health care and social service model. To understand any implementation challenges and prospects for the program from the perspective of stakeholders prior to full implementation, key informants from several relevant sectors were interviewed in the fall of 2017, before the monthly care coordination payments began.

Requests for interviews were sent to associations representing IDD and BH providers, associations representing IDD and BH beneficiaries, the Arkansas Hospital Association, the Arkansas Pharmacy Association, and representatives of the five PASSEs that submitted letters of intent to join the program [34]. The provider associations were identified as key informants because PASSE ownership and initial network adequacy required BH and IDD providers, physicians, hospitals, and pharmacies.

The interview consisted of a 12-question instrument (supplementary Fig. 1) that addressed issues of potential costs, quality, access, and satisfaction with PASSE, and any implementation challenges. In the final two questions, informants had the opportunity to voice any other opinions about the program, and to distill a message they thought local and state policymakers should hear. Informants were given a range of options regarding confidentiality, ranging from total confidentiality to willingness to share their names and their responses. Hand-written notes taken during the interviews were analyzed and coded by themes.

**Fig. 1:**
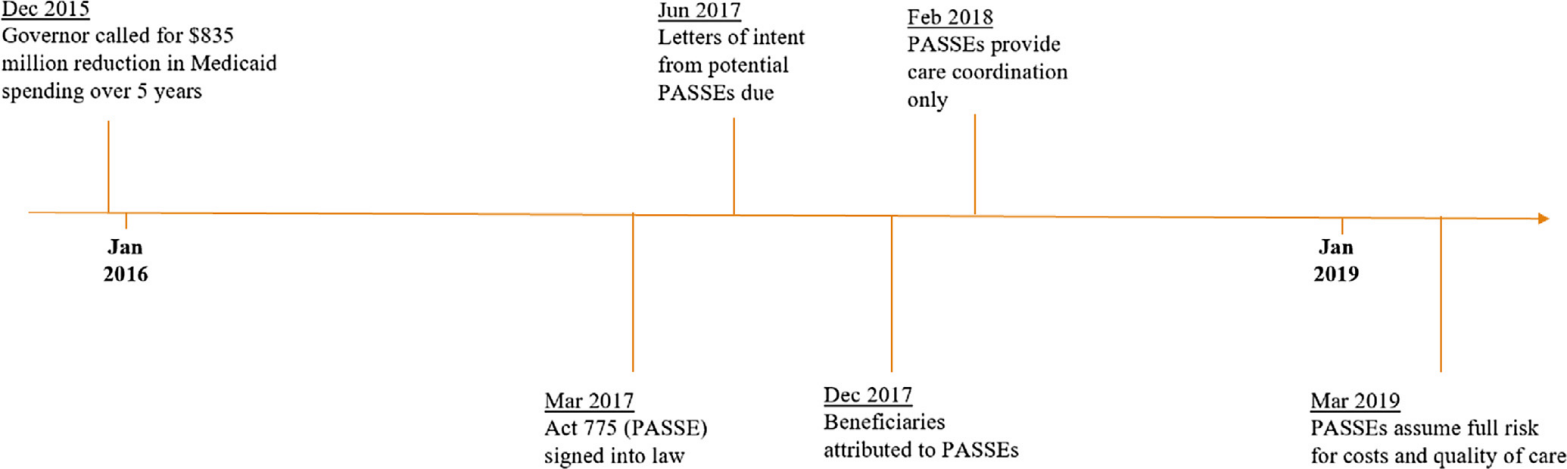
Timeline of PASSE Implementation.

Thirteen requests for interviews were sent; ten interviews were completed. An in-person interview was not feasible for one informant; two informants did not respond to the request for an interview. Interviews were conducted in person and lasted no more than one hour. Representatives from all five prospective PASSEs completed interviews. Four of the seven informants representing the PASSEs also represented BH or pharmacy associations. The remaining three informants represented the state hospital association, beneficiaries with IDD, and children and families.

## 3 Results

### 3.1 Implementation

PASSE implementation proceeded rapidly, with twenty-three months from legislative passage to full implementation; see Fig. 1 for a timeline of implementation. Act 775 was executed on March 31, 2017; letters of intent from prospective PASSEs were due three months later, in June 2017. The independent assessments of beneficiary tier level began while the Arkansas Insurance Department was in the process of licensing PASSEs. Key informants representing PASSEs reported difficulty locating beneficiaries as providers often did not have current contact information, particularly for beneficiaries with BH conditions. Indeed, assessors encountered the same problem in trying to conduct assessments. Instead of relying solely on mail and telephone outreach to locate beneficiaries, assessors shifted tactics and stationed themselves in BH provider offices, conducting assessments on site as beneficiaries arrived for scheduled or walk-in appointments. Informants expressed concern that assessors were not required to have a clinical background and questioned the accuracy of some assessments.

Once tier level was determined and a beneficiary was attributed to a PASSE, DHS provided the PASSE with three years of claims history for the beneficiary. Shortly thereafter, on February 1, 2018, PASSEs began to receive monthly payments in return for providing care coordination for attributed beneficiaries. PASSEs were to use this time, during which claims continued to be reimbursed fee-for-service, to analyze the claims history of attributed beneficiaries and gain experience providing care coordination without being at-risk for all Medicaid services. Several informants reported problems with the accuracy and timeliness of data provided by DHS, potentially limiting the usefulness of the lead time.

By April 1, 2018, PASSEs had to demonstrate an adequate, statewide referral network of BH providers, IDD providers, hospitals, physicians, and pharmacists. Network adequacy is defined for each provider type by a maximum distance from each beneficiary in an urban or rural area and by a maximum ratio of providers to beneficiaries. DHS officials and stakeholders reported that PASSEs struggled to develop their contracted networks, particularly with non-BH or IDD providers. In part due to the rapid implementation timeline, providers around the state remained unaware of the program. In addition to the limited time for implementation, informants pointed to inadequate provider and beneficiary outreach, education, and training on the new model. As program implementation approached, DHS increased education through town hall meetings, webinars, and training materials, and published a provider manual [35], [36]. Nevertheless, the lack of familiarity, combined with uncertainty of how to assess their financial risk, left providers hesitant to join PASSE networks. Full risk was scheduled to begin January 2019 but was extended to March 1, 2019 [37], due in part to system testing and to the issues with network recruitment. The struggle has continued through the next round of network adequacy assessments, when PASSEs were required to meet 70–80 percent of network standards, depending on the type of provider, before assuming financial risk for all Medicaid services [23]. However, once DHS announced there would be no further implementation delays, the PASSEs reported large increases in provider contracting. A transition period of graduated network adequacy compliance was observed until September 2019. Additionally, open enrollment was delayed from May to October 2019 to permit beneficiaries to make a more informed choice of a PASSE based on their network [38]. The PASSEs now must meet full network adequacy standards for all provider types [23]. See Table 5 for a summary of lessons learned from implementation.

**Table 5:** Lessons Learned from PASSE Implementation.

**Challenges**	**Solutions**
Locating BH beneficiaries to conduct independent assessments	• Along with standard mail and phone outreach, station assessors in BH provider offices to conduct assessments with beneficiaries present for scheduled or walk-in appointments
Conducting adequate provider and beneficiary outreach, education, and training	• Increase provider and beneficiary education through town hall meetings, webinars, and training materials • Publish a provider manual
Developing PASSE networks	• Two-month implementation delay • Communicate firm, non-negotiable implementation date after implementation delay • Allow graduated network adequacy compliance post-implementation • Six-month delay in open enrollment

Within the short implementation timeline, DHS issued little guidance related to PASSE operations. PASSEs have had relative freedom to select their business partners, develop provider networks, and establish care coordination processes. Five letters of intent were submitted by prospective PASSEs. As implementation proceeded and the PASSE owners learned what it would take to succeed in the program, not all remained in operation. One informant noted the feeling of “flying the plane while we are building it.” DHS had anticipated that two or three PASSEs would have sufficiently large networks, resources, and number of beneficiaries to be financially viable. Indeed, four of the five progressed to beneficiary attribution, and three are currently in operation and have begun to accept full risk for all Medicaid services. The limited guidance and communication from DHS were a frustration for many informants interviewed and may also have contributed to the decline in the number of PASSEs.

DHS executed a series of waivers, waiver amendments, and state plan amendments to implement PASSE [13]. A Section 1915(b) waiver authorized the establishment of a Primary Care Case Management entity to cover the period between February 2018 and March 2019, when the PASSEs provided care coordination only. A second Section 1915(b) waiver, effective March 2019, authorized the full-risk model. Conforming amendments under Section 1915(c) authority made the necessary changes for beneficiaries who previously would have received services under the state Medicaid IDD waiver. A 1915(i) state plan amendment expanded community-based services. To monitor and oversee PASSE implementation, operations and services, DHS created a new division, the Office of Innovation and Delivery System Reform [39]. The office will be responsible for collecting beneficiary self-report data (e.g., CAHPS), analyzing non-claims data such as disenrollment requests, conducting focused studies on topics such as coverage of services and quality of care, geographic mapping of PASSE provider networks, conducting initial and annual on-site reviews, leading performance improvement projects, and looking for provider outliers.

### 3.2 Future prospects

Overall, informants described the potential for the design elements of PASSE to stimulate changes in provider and beneficiary behavior. Following is a critical appraisal of the program based on potential benefits and concerns articulated by the key informants and summarized in Table 6.

**Table 6:** Potential Benefits and Concerns for PASSE from Key Informants.

**Potential Benefits**	**Concerns**
• Care coordination • Flexibility to provide services not covered in state Medicaid program • Incentive to make community investments • Accountability for costs and quality of physical, behavioral, and long-term care • Challenge to meet quality targets • Fostered competition • Provider ownership • Capitation & shared savings	• Lack of provider behavior change • Separate systems for more and less complex beneficiaries • Managed care stinting • Administrative burden of interacting with different PASSEs

#### 3.2.1 Potential benefits

One of the benefits mentioned most often by key informants and echoed in the literature is that PASSEs will have the opportunity to improve beneficiary outcomes by fostering coordinated, integrated care, a model that has shown clear benefits for individuals with BH conditions [40] and promising results for individuals with IDD [41], [42], [43]. PASSE advances coordinated care through the inclusion of accountability for physical, behavioral, and long-term care within capitation payments. Care coordination, and the accompanying focus on a person-centered service plan, has long been a benefit for beneficiaries with IDD, but will be a new and welcomed benefit for beneficiaries with BH conditions.

The expanded definition of care coordination, the flexibility to provide services not covered by the state Medicaid fee-for-service program and the incentive to make community investments also foster coordinated care by widening the scope of accountability for the lives of beneficiaries. About three quarters of the key informants felt that with the increased flexibility and accountability for physical, behavioral, and long-term care providers would begin to work together to address the needs of the whole person, versus, as one informant stated, “What can I bill?” DHS staff report that the program has already seen some benefits of improved coordination, for example in greater scrutiny of institutional placement of children in foster care and in efforts to expedite behavioral health treatment for offenders involved in state juvenile and adult judicial systems. Several other states have begun to formally recognize the influence of social determinants of health on spending and outcomes by including requirements to address determinants like employment, criminal justice, and child care in their Medicaid contracts [44].

Several key informants representing PASSEs and provider associations cited PASSE accountability for meeting quality targets as a promising mechanism to improve beneficiary outcomes. One informant expressed confidence that the accountability would “bring quality to Medicaid like in the private industry.” Pay-for-performance models in health care have shown inconsistent associations with quality measures, with positive changes mostly in process and intermediate outcome measures [45]. Despite this mixed evidence, payment models not tied to quality may present a greater risk of achieving cost savings at the expense of quality. Both the contractual requirements to meet certain, basic access and quality targets as a condition to remain in operation, and the quality incentive payments available for meeting additional, to-be-determined targets are expected to deter a decline in quality of care and promote improvements in beneficiary outcomes. The public reporting of PASSE quality metrics is also expected to improve beneficiary outcomes through competition fostered by the choice beneficiaries have between PASSEs. As another informant noted, “Free markets will work.” DHS officials have pointed to existing local monopolies of BH and IDD providers as contributing to poor quality of care. PASSE owners have the freedom to choose their partners and to build their networks as broadly or narrowly as desired. In this way, along with the initial attribution methodology, the program is indeed provider-led and will initially favor existing beneficiary-provider relationships. Over time, though, beneficiaries will have access to quality measures for each PASSE and the annual option to select a different PASSE. To attract members, PASSEs may recruit new providers as a route to improving access to and quality of care. The competition fostered by these conditions is expected to lead to an expanded pool of specialty providers, reduced need for institutional placement, and more patient-centered care [46].

All key informants representing provider associations or beneficiaries expressed their preference for provider ownership of the PASSEs instead of a traditional managed care organization, while noting that a provider-led model is “a difficult way to do it.” The requirement for PASSEs to be provider-owned draws upon evidence suggesting a benefit of provider leadership of organizations seeking to manage and change the behavior of provider groups. Among organizations managing provider groups, those with provider leadership have been associated with greater diffusion of innovation within the provider groups [47] and better patient ratings of access to and quality of care [48]. Provider leadership may encourage organizations to achieve these outcomes by creating a culture that values open communication and transparency with provider groups and the patients they serve [49], and by promoting cooperative relationships between the organization and provider groups that signals to the provider groups that their views are represented in organizational decision-making [50]. Trust engendered by provider leadership in the PASSEs may be key to promoting the practice change necessary to improve quality and reduce spending. The blended design – requiring both provider ownership and risk management experience - combines the success of ACOs in reducing costs and improving quality of care [51], [52] and the managed care benefit of shifting financial risk from state budgets. Blended designs hold promise to avoid some of the pitfalls of earlier MCO efforts to shift financial risk to providers by paying them via capitation for a defined population. Providers had little experience with risk management, often lacking a sufficiently large patient population to adequately spread the risk while remaining profitable, and many failed [53]. A blended design is one in which the accountable organization consists of both providers, who have the patient care experience needed to effectively coordinate care, and entities with experience managing financial risk.

Most key informants cited the capitation payment structure as the primary mechanism for achieving the cost savings called for by the Governor. They expected DHS would set the capitation payments at a level that achieved most of the required savings off the top. Informants representing PASSEs or provider associations pointed to the opportunity to earn shared savings as the secondary mechanism for cost savings. Profit maximization is intended to incentivize the PASSEs to reduce costs to a point that maximizes shared savings while achieving sufficient quality to, at a minimum, comply with contract requirements and to attract an adequate number of members to stay in business, and at best earn all available quality incentive payments. Informants expressed hope that it would be through care coordination, service flexibility, and provider leadership that PASSEs achieved the spending reductions necessary to earn shared savings.

#### 3.2.2 Concerns

One of the planned benefits of PASSE was the considerable lead time PASSEs had to examine the claims history of their beneficiaries prior to bearing full risk for all services. PASSEs were to use the time between attribution and full-risk capitation payments to gain a more complete understanding of their risk pool and to begin to deliver intensive care coordination to prevent future costly care (e.g., hospitalizations). Despite DHS training, to date it appears PASSEs are continuing to operate much the same as providers have in the past by, for example, simply checking in with beneficiaries once a month to assess satisfaction with services. PASSEs are required to report any alternative payment arrangements with providers, but none have been reported to date.

PASSEs may be anticipating that little to no change in practice will be required. Included in this assumption is that most Tier I beneficiaries will join the program when able and select the PASSE to which their current long-term care provider belongs, allowing providers to maintain the same patient population. Two key informants representing PASSEs and provider associations expressed concern that limiting required enrollment to Tier II and Tier III beneficiaries may hinder the ability of a PASSE to manage risk. If Tier I beneficiaries remain in traditional Medicaid, the dual system may threaten both the sustainability of PASSE and the quality of care for beneficiaries, as the same pool of providers must follow requirements for two programs (PASSE and traditional Medicaid). The PASSEs may view this scenario as untenable and therefore unlikely to occur and continue with business as usual. However, the lack of evolution in provider practices may portend that the key care coordination component of PASSE will not fulfill the quality-enhancing and cost containment potential envisioned. The present period of transition to full risk and graduated network adequacy requirements, and the upcoming start of voluntary Tier I enrollment will reveal more about if and how the approach to services will change.

Almost all key informants had concerns that there would be stinting on care due to the shift from fee-for-service to capitated payment. Under the former structure, providers profit from increasing spending, while under the latter the accountable organization profits from reduced provider spending, which can risk deterioration in health and quality of life. Most evaluations to date indicate greater negative than positive quality of care effects of Medicaid MCOs for people with BH conditions or IDD [54], [55], [56], [57], [58], [59], [60]. Compared to fee-for-service or PCCM, managed care models for Medicaid beneficiaries with BH conditions were associated with increased preventive care [54], but poorer health and greater perceived unmet mental health needs [55]. Beneficiaries with IDD in managed care have reported problems with transportation, communication, and the inability to talk to a care coordinator [60], while family members have seen both positive (e.g., improved access to care and care coordination) and negative (e.g., worse quality of care and quality of life) changes after managed care implementation [58]. The absence of independent, or conflict-free case management may contribute to the reports of decreased quality of care under managed care, as family members perceived that service coordinators, as employees or contractors of an MCO, were no longer advocates for the beneficiary [58]. The evidence also suggests that Medicaid MCOs have not decreased state spending [61], including spending for beneficiaries with disabilities [62], [63]. The lack of savings may be the result of already low reimbursement rates, of states already having implemented some degree of utilization review, prior authorization, and other methods to control utilization before turning to managed care, or from the expense of establishing the administrative infrastructure to contract with and regulate the MCOs [61].

The considerable evidence of negative impacts of managed care for people with BH conditions or IDD raises questions of how PASSE beneficiaries will fare. Already a vulnerable group because of the complexity of their disabilities, PASSE beneficiaries face the added challenge of living in a high poverty, predominately rural state, perhaps rendering their well-being more sensitive to changes in the quality of Medicaid services than beneficiaries in other states. If the blend of provider and risk management leadership in the PASSEs does not lead to sufficient cost savings in short order, key informants worried that the state may modify the program to resemble a traditional MCO, with one informant noting “providers are scared to death” of managed care. Regardless of the model, some key informants representing provider associations cited the additional administrative burden of having to interact with different managed care policies and procedures, billing systems, etc.

## 4 Discussion

Arkansas has blended promising elements of previous and current U.S. payment reform models in designing PASSE. Accountability for acute, behavioral and long-term services and for the coordination of non-Medicaid services, quality targets tied to payment, statewide competition for members, and the capitation payments with the opportunity to share in savings all portend positive quality of care outcomes and an absence of spending increases, if not spending decreases. However, the undesirable cost and quality outcomes of previous managed care arrangements for people with BH conditions or IDD, and the risk ambiguity presented by the dual payment systems for these populations suggest that quality improvements and spending reductions are far from certain.

The success of PASSE will be challenging to quantify. PASSE administrators must establish protocols for monitoring quality of care and beneficiary outcomes and are striving to select measures that are meaningful to beneficiaries and can identify improvements or deteriorations in quality of life. Researchers, policymakers and others have identified a lack of consensus about how to assess the quality of care delivered to people with chronic conditions, and have called the gap in a meaningful evaluation strategy a “significant limitation” to monitoring the impact of Medicaid for beneficiaries with disabilities [64], [65]. The lack of consensus has led program evaluators to rely on measures of access, processes of care, and clinical outcomes, even though improvements in these metrics often do not translate to improvements in long-term, patient-valued outcomes like quality of life [45], [66], [67]. Organizations such as the Patient-Centered Outcomes Research Institute [68], CMS [69], and the National Council on Disability [70], and groups representing researchers, clinicians, and service users [71], [72], [73] have recommended that service users be the primary decision makers in selecting outcomes to assess the success of health and social services. Selecting the appropriate quality metrics and measurement strategy will be critical in evaluating the impact of PASSE, particularly to capture the life course outcomes of young beneficiaries.

## 5 Limitations

We are aware of several limitations of the paper. First, since PASSE is new, we are not able to present outcomes of the program. The purpose of the present study is to describe how PASSE fits within the context of Medicaid programs for beneficiaries with complex needs, and to understand the prospects for the program from the perspective of the individuals and organizations responsible for making it work. Rigorous evaluation with appropriate methods and measures of access, process, satisfaction, and outcomes will be an important next step to understand the mechanisms and effect of this type of systems change. Second, the key informants interviewed offered a particular perspective. A different set of informants may have seen different prospects for the program. The informants were chosen based on their knowledge of PASSE, their involvement in the development of PASSE, and their prominent role in determining the success of PASSE. Third, in the environmental scan of state Medicaid programs for beneficiaries with BH conditions or IDD, we may have missed relevant programs in the review of public documents and webpages.

## 6 Conclusion

PASSE architects have drawn from the promising elements of previous payment reforms to create a unique model of care for Medicaid beneficiaries with BH conditions or IDD. Holding each PASSE accountable for the physical, behavioral, and long-term care needs of attributed beneficiaries, directing attention to social determinants of health, requiring PASSEs to be provider-owned and include an entity with risk management experience in the ownership structure, requiring statewide coverage for each PASSE, and establishing shared savings and pay-for-performance incentives are all features anticipated to bring down costs and improve quality of care for beneficiaries. Conversely, there are concerns that shifting to a managed care structure and focusing only on beneficiaries with the most complex needs may have a negative effect on beneficiary outcomes. Will providers continue with business as usual, or will PASSE succeed in aligning the incentives to reduce spending, improve outcomes, and maximize profit? As Arkansas continues to roll out the PASSE program, other payers such as CMS, other states, commercial insurance companies, and other countries will be keen to see whether payment reform for the most vulnerable and expensive beneficiaries can achieve improvements in costs or quality.

## Funding sources

The research reported herein was performed pursuant to a grant from the U.S. Social Security Administration (SSA) funded as part of the Disability Research Consortium. The opinions and conclusions expressed are solely those of the author(s) and do not represent the opinions or policy of SSA or any agency of the Federal Government. Neither the United States Government nor any agency thereof, nor any of their employees, makes any warranty, expressed or implied, or assumes any legal liability or responsibility for the accuracy, completeness, or usefulness of the contents of this report. Reference herein to any specific commercial product, process, or service by trade name, trademark, manufacturer, or otherwise does not necessarily constitute or imply endorsement, recommendation or favoring by the United States Government or any agency thereof. Additionally, the third author was funded by a Robert Wood Johnson Foundation Health Policy Fellowship.

## Declaration of Competing Interest

The authors declare that they have no known competing financial interests or personal relationships that could have appeared to influence the work reported in this paper.
