# Supplementary material for: Risk-based contracting for high-need Medicaid beneficiaries: The Arkansas PASSE program
Source: Health Policy Open. 2020 Dec 3;2:100023. doi: 10.1016/j.hpopen.2020.100023 (PMC10297806; doi:10.1016/j.hpopen.2020.100023)
Supplement: Supplementary data 1 [file mmc1.docx]

**Supplementary Figure 1** Key Informant Interview Questions

1. Describe your experience with the current Fee For Service Medicaid system.
2. Describe your experience with Act 775 Provider-Led Arkansas Shared Savings Entity.
3. How does Act 775 impact you or your organization?
4. What difficulties have you experienced with the implementation of Act 775?
5. How do you think this Act will improve the quality of healthcare in the tier 2 and tier 3 BH/DD special population?
6. How do you think this Act will **not** improve the healthcare of the tier 2 and tier 3 BH/DD special population?
7. Do you think the State of Arkansas will achieve cost savings with this approach? Why or why not?
8. Do you think the tier 2 and tier 3 BH/DD special population will have greater access to healthcare?
9. What will be the level of satisfaction among providers/consumers with the PASSE model?
10. If Act 775 fails, then a third party MCO will be implemented. Describe your thoughts/opinion on this. Do you feel it would be better or worse than the PASSE?
11. Do you have anything else you want to share that you haven’t had a chance to?
12. What is one message you would send to our local and state policy makers on this topic?
